# Editorial: Stem cells and extracellular vesicles in aging-related diseases

**DOI:** 10.3389/fcell.2024.1467532

**Published:** 2024-09-18

**Authors:** Bin Jiang, Li Duan, Junjun Li, Li Yan

**Affiliations:** ^1^ R&D Division, Eureka Biotech Inc., Philadelphia, PA, United States; ^2^ Department of Orthopedics, Shenzhen Second People’s Hospital, The First Affiliated Hospital of Shenzhen University, Shenzhen, Guangdong, China; ^3^ Department of Applied Physics, Osaka University, Osaka, Japan; ^4^ Fischell Department of Bioengineering, University of Maryland, College Park, MD, United States

**Keywords:** stem cells, aging-related diseases, extracellular vesicles, disease model, cancer

Experiencing age-related diseases such as stroke, osteoarthritis, and diabetes can be profoundly challenging. Current treatments involving pharmaceuticals and surgeries often need to catch up regarding efficacy, cost, and applicability. In contrast, stem cells and extracellular vesicles (EVs) present a promising alternative, as their effectiveness and applicability have been validated in animal models. Here, we are excited to summarize the latest papers on this Research Topic for the audience.

Stroke in the elderly is a significant health concern, often leading to severe disability or death and necessitating prompt medical intervention and comprehensive rehabilitation. Treating stroke in elderly patients is complicated due to preexisting health conditions, reduced recovery capacity, and mobility issues. Li et al. report a novel approach using mitochondria derived from healthy, youthful mesenchymal stem cells (MSCs) to protect neurons in a mouse model of ischemic stroke. Their data suggest that MSC-derived mitochondria hold potential for translational applications as a treatment for ischemic stroke patients. Their work gives us a new insight into biomedical resources like organelles between stem cells and EVs.

In the rapidly aging U.S. population, age-induced bone diseases such as senile osteoporosis and osteoarthritis represent major public health concerns. These conditions significantly increase the risk of low-trauma fragility fractures, which are debilitating, cause significant morbidity and mortality, and are costly to treat and manage. Liu et al. discuss a new concept of osteoimmunology, which involves molecular and cellular crosstalk between the skeletal and immune systems. They also review osteogenesis and its differentiation, focusing on distinct Toll-like receptors (TLRs) variants on MSCs from various tissues and detail the impact of TLR pathway activation or inhibition on MSC osteogenic differentiation and elucidate the interplay of TLR pathways with osteoclasts, immune cells, and MSC-derived EVs. Moreover, Bregowda et al. identify alpha-2-macroglobulin (A2M), a pan-protease inhibitor that binds inflammatory cytokines, as a novel target in osteoporosis. They found that downregulation of A2M in bone marrow promotes skeletal stem/progenitor cell (SSPC) dysfunction and imbalances in skeletal homeostasis.

The continuous use of various medications significantly impacts our quality of life. Many reproductive-aged men require long-term drug therapy for conditions such as cardiovascular disease, cancer (including chemotherapy and radiotherapy), hyperlipidemia, and depressive disorders. Mo et al. review the influence and mechanisms of chemical medications on male fertility, as well as the progress of stem cell and exosome therapies for male infertility. Their review aims to provide recommendations for evaluating the effects of drugs on male fertility, both positive and negative, in clinical applications and to offer strategies for the diagnosis and treatment of male infertility. The review also highlights the need for further research into the underlying mechanisms and potential therapeutic targets to improve male reproductive health.

Following the drug influence on life quality mentioned above, more chemicals and cytokines significantly impact stem cells’ performance and their derived EVs. For instance, MSCs respond to inflammatory environments and stimuli such as Tumor necrosis factor-alpha (TNF-α) and Interleukin-8 (IL-8) by secreting anti-inflammatory cytokines like IL-10 and IL-11. These cytokines help to reduce inflammation and prevent excessive immune responses that can cause tissue damage, such as in late-stage sepsis. One strategy to enhance the efficacy of MSCs after transplantation is to prime them with inflammatory cytokines, thereby preparing them for the *in vivo* environment, such as the joint cavity of the knee. Xia et al. found that MSCs primed with IL-1β showed decreased proliferation and migration rates but enhanced chondrogenic differentiation capacities. Furthermore, various MSCs demonstrated efficacy in restoring damaged articular chondrocytes *in vitro*.

In addition to cytokine priming, artificially synthesized chemicals can also augment MSC performance in conditions like diabetes mellitus and retinal pericyte loss. Lu et al. generated a novel lipid mediator, 7S,14R-docosahexaenoic acid (7S,14R-diHDHA), a maresin-1 stereoisomer biosynthesized by leukocytes and related enzymes. This compound has inflammation-resolving and protective properties. Moreover, 7S, 14R-diHDHA can enhance MSC functions in ameliorating diabetes mellitus and retinal pericyte loss in diabetic db/db mice.

Although EVs are promising for treating age-related diseases, their practical applicability requires further investigation due to their inherent properties and practical limitations. For instance, while some research suggests that EVs could be used for cancer treatment based on animal model experiments, translating these findings to clinical practice in patients remains challenging. However, EVs offer significant potential for early cancer diagnosis and prevention. Wang et al. provide an in-depth analysis of the role of EVs in cancer, discussing their advantages and limitations for both treatment and diagnosis. Their review includes a comprehensive overview of the latest technological innovations involving exosomes, emphasizing their potential to enhance the efficacy of early cancer detection. These insights highlight the need for continued research to fully understand and harness the capabilities of EVs in clinical settings, particularly for age-related diseases and cancer.

In conclusion, stem cells and EVs offer a gentle and effective option for treating aging-related diseases. There is a consensus that these therapies, including other stem cell-derived materials like matrices and cell organelles, are complementary and beneficial for treating these conditions. More research on stem cells and their derived EVs is essential for understanding the mechanisms and developing innovative therapeutic strategies and molecular targets for preventing and treating aging-related diseases.

